# Impact of the three waves of COVID-19 pandemic on the HR practices of Hungarian organizations–Experience from an empirical study

**DOI:** 10.1371/journal.pone.0283644

**Published:** 2023-06-09

**Authors:** Krisztina Dajnoki, Beáta Sz. G. Pató, András István Kun, Erika Varga, Arnold Tóth, Botond Géza Kálmán, Ildikó Éva Kovács, Szilvia Szabó, Katalin Szabó, Zoltán Majó-Petri, Lóránt Dénes Dávid, József Poór

**Affiliations:** 1 Faculty of Economics and Business, University of Debrecen, Debrecen, Hungary; 2 Faculty of Social Sciences, Eötvös Loránd University, Budapest, Hungary; 3 Hungarian University of Agriculture and Life Sciences, Szent István Campus, Gödöllő, Hungary; 4 Faculty of Finance and Accountancy, Budapest Business School, Budapest, Hungary; 5 Institute of Business, Budapest Metropolitan University of Applied Sciences, Budapest, Hungary; 6 Faculty of Economics and Business Administration, University of Szeged, Szeged, Hungary; 7 Institute of Rural Development and Sustainable Economy, Hungarian University of Agriculture and Life Sciences, Gödöllő, Hungary; 8 Faculty of Economics and Business, John von Neumann University, Kecskemét, Hungary; 9 Faculty of Economics and Informatics, J. Selye University, Komarno, Slovakia; Universiti Pertahanan Nasional Malaysia, MALAYSIA

## Abstract

Over recent decades, the practice of human resource management in the transitional countries of Eastern Europe and in Hungary has changed significantly. Especially in local subsidiaries of foreign-owned companies and in the leading domestic large organizations, HRM has become a strategic function, while in the practice of small and medium-sized enterprises it is less common. COVID-19 hit companies, institutions and individuals unexpectedly, not only in Hungary but also in the more developed regions of the world. This crisis has also highlighted the fact that larger and better prepared organizations and public institutions have found it easier to weather this global human catastrophe. We analyze how the key tasks of HRM have changed during the successive waves, along four hypotheses. Initially, health protection, communication and home-office organization were the focus of the work of human resource professionals. In the second and third waves, securing and retaining staff became more important.

## Introduction

Human resource management plays different roles–operational, tactical, service, strategic–in the life of organizations, with a variety of tasks [1, 2]. In one single blow, the pandemic has superseded the role and responsibilities of HR in the life of organizations, and HR has had to adapt, adjust and in many cases provide new solutions [3, 4]–even stepping out of its existing role–for an unfamiliar situation. The pandemic has further emphasized the ’human’ element of HR [5, 6]. The different HR roles have shifted with the pandemic, and a new mix is beginning to emerge, where operational, tactical and strategic roles are superseded by the pandemic and a ’hybrid’ HR picture of a crisis manager dynamically adapting to the pandemic situation is beginning to emerge [7, 8].

The main question of own research is how does the human resource management practice of the Hungarian corporate/institutional sphere respond to the challenges of the crisis caused by the coronavirus in the first, second and third waves of the pandemic? The aim of the article a literature overview of the impact of COVID-19 from a socio-economic and HR perspective particularly HR-related experiences of crisis management and crisis resolution. Basis on the references we present our hypotheses on the subject. Our aim is to present the results of the main components and characteristics of our statistical analysis related to our four hypotheses. Our objective is basis on the results of own survey, formulate conclusions and proposals that can be used by other organizations.

Pandemics are unique among crises because they attack human capital, i.e. the health of the population [9]. However, before COVID-19, there was no pandemic in economic history that forced almost every country in the world to shut down its economy on such a scale [10, 11]. In the pandemic period, the focus of HRM shifted to health and safety [12], because economically developed countries have reached a stage where effective economic growth is no longer based on additional capital investment in the production process, but on added human value, so-called human capital. The crisis was due to the fact that the fear of infection and the restrictions on absenteeism from work imposed by the pandemic have reached a social scale. Not only educational institutions but also productive enterprises have shut down [13]. In the past, overproduction, over-crediting and large income disparities typically triggered a global crisis and a general recessiondue to the failure of the financial system [14], but now global shutdowns have made scarce reproduction permanent.

COVID-19 has also revealed significant labour market inequalities. These include gender inequalities [15] or income inequalities, which ultimately affect workers personally, and therefore HR has a key role to play in addressing them [16]. HR professionals, researchers and trainers should seek solutions to effectively address the problem. For this to happen, HR research itself will have to change [17]. The restrictions also had an immediate impact on the labour market, with increased unemployment and more frequent teleworking or reduced working hours. Many workers saw their working hours and wages reduced, and many were made redundant. Lay-offs were mainly a threat for workers who are unable to perform at least 40 per cent of their tasks through teleworking [18]. These workers, and those with temporary or fixed-term contracts, were the most affected, while others were sent home to telework. This raises the question of the extent to which HR can be held responsible for these inequalities. On the one hand, it can be shown that HR is responsible, despite all its efforts, for inequalities that are the result of a failed HR policy.

Meanwhile, it is not only workers’ incomes that have fallen. The production stoppage led to a 3.5 per cent decline in real global GDP in 2020 [19, 20]. The COVID-19 crisis hit Hungary’s export-oriented economy hard, ending a period of steady growth in 2016–2019, during which incomes grew steadily and the unemployment rate fell to a 30-year low (3.4%) [21]. (In Hungary, government-level measures are primarily aimed at protecting health and jobs [22, 23]. At company level, these are the responsibility of HR. During both the spring and autumn waves, a large-scale company-level survey on this topic was conducted in Hungary. In addition to the usual and established measures, new solutions have emerged and even taken central roles, which HR professionals have explicitly linked to the pandemic as a root cause. This also indicates that the exceptional situation caused by the pandemic has called for a structural change [24], new HR policies and regulations to reduce workload and improve the quality of work. Such regulations can create practices that manage human resources appropriately, ensuring job security, adequate working conditions and employee welfare [25]. In addition, classic layoffs, previously most commonly used as a first response, can also be traced in the literature. Initially, they mainly affected the hotel [26] and restaurant [27] sectors, mobile and other hospitality, passenger transport [28], the electrical equipment and appliance sector [29], and the metalworking sector, followed by accounting, auditing and tax consultancy [30]. In Hungary, the biggest loss-maker was commercial accommodation, which reported a 99% drop in overnight stays and a 97% drop in revenues in April 2020 [31]. The situation in the sector had only partially recovered by August 2020.

Not all companies and organizations have been affected equally by the global crisis. There have been winners alongside the losers. However, job creation or job growth occurred in far fewer areas: most of them in e-commerce-related IT [32, 33] and logistics jobs [34]. Restrictions have led to a shift of trade to the online space, making many retail sales, cashier and distribution jobs redundant [35].

Many businesses and organizations had pre-defined plans for dealing with different emergencies or addressed pandemic preparedness in their business continuity plans, but few have recently assessed and updated these plans. However, COVID-19, although unexpected by almost everyone, is not a classic black swan event [36, 37].

The human resource-intensive nature of the pandemic has also brought health-related HR activities to the fore. Teleworking is also a perfect solution in this respect, as the home worker is protected against workplace infections. In the first wave of the UK outbreak, 80 percent of those infected were health and social care workers [38], i.e., jobs which can only be done by attendance at the workplace. According to Spanish research data, work involving a physical presence at the workplace increased the risk of infection by 76% [39], mainly in confined spaces–i.e. in 92% of workplaces.

Governments in different countries, including Hungary, recognized relatively quickly that the poor and the less skilled workers would be most affected by this global crisis. After the first wave and the second wave, it became clear that the pandemic would lead to a very significant economic contraction of 4–6% worldwide, with the exception of China (+2%) (COVID-19, 2020). However, the massive vaccination campaigns that have been launched in the meantime have led to a glimmer of light at the end of the tunnel–the possibility of 4–6% growth following the downturns [40]. [41] propose three different scenarios for recovery–in other words, for the process of social disconnection–in the context of the COVID-19 crisis:

- *Let’s get back to normal*, when we take the line that we must quickly stop defending against the virus, and open up the economy. Representatives of this line often criticize governments for what they see as unnecessary pandemic controls.
- *There’s no going back to normal* means that new regulations must be put in place to prepare society to prevent a similar global pandemic from breaking out.
- *Let’s strive for a new and better normal* is based on the experience that COVID-19 has led to a high level of cooperation and collaboration in different areas of socio-economic life on which new, previously unused regulations can be built.

In the wake of the second and third waves of the pandemic, there is a growing recognition that slowing down COVID-19 or removing restrictions is “not about returning to the pre-pandemic period, but rather gradually and cautiously changing to a new norm, while being ready to reintroduce measures if they are needed again” [42]. Various experiences show that where HR has been able to address problems in a strategic and complex way, they have been able to make the transition more quickly [7, 43]. The pandemic has also drawn attention to the fact that HR can do much to address inequalities [16] and to protect workers’ health more consciously [44].

The recent second and third waves of COVID-19 have increasingly highlighted that changes have also affected employees’ relationships with their employers. We are thinking here of the workplace insecurity, anxiety and depression triggered by the pandemic [45] the relocation of the former workplace to the employee’s home (working from home) [46, 47] or the digitalization of workplace interactions (e.g., meetings, discussions, etc.) such as teleconferencing through Zoom or Teams. The insecurities caused by the pandemic have led to more frequent job changes. In this new context, the need for more flexible working arrangements has been reinforced by employees [48].

Basis on literature review, we set up the following hypotheses. After each hypothesis, we present its grounding in the literature.

### H#1: Crisis management measures in the HR area will reposition the importance of individual HR functions

The importance of this issue has been highlighted by the following authors. [49] focuses on crisis management planning to retain and protect the workforce, and [50] on education and training. Meanwhile, not only past and present but also future possibilities have been analyzed [51]. [52] highlight a rapid and flexible response, tailored measures, carefully selected staff, appropriate situation analysis and communication as the basic conditions for effective crisis management. [53] cite reducing working hours, managing employee stress, flexible HR measures and the provision of protective equipment as key tasks, although they do not mention the possibility of reducing staffing levels in relation to cost reduction. The pandemic has led to the reinvention and further development of old solutions [54]: the paradigms of teleworking, digitalization and automation have been renewed. The changes had to be implemented quickly, which also transformed the role of management: transparency and trust were valued. [55] discusses ways of retaining staff (wage cuts, restructuring, flexible working conditions, reduction of other costs). He stresses that the guarantee of success is strong management with clear and unambiguous objectives.

### H#2: The most commonly used crisis management measures in HR are organizational context-dependent

#### H#2a) The crisis management measures used depend on the size of the organization

According to [5], pandemic-induced changes in all areas of HR strategy (health, recruitment, communication, mobility, training) were proportional to the size of the organization. In other words, small firms experienced little or no change in HR operations, while large firms experienced significant changes in all areas. This may be partly related to the finding [56] that only one in ten employees in the largest firms (over 10,000 employees) had a serious fear of losing their job. In the small and medium-sized enterprise sector, the figure is almost 30%, and it is 66% among the self-employed. The reason for firm size being a determinant of crisis policy is probably that larger firms are more capital-rich and therefore able to survive longer crises [57]. Another possible reason is that firm size is also associated with greater job security. This effect was reinforced by the fact that initial policy responses did not provide sufficient protection for workers in small and medium-sized enterprises. These factors may explain why the HR measures implemented depended on firm size: for example, 57% of large firms, 51% of medium firms and 47% of small firms have introduced mandatory mask wearing in confined spaces, and a quarter of large firms plan to require vaccination for work, compared to a third of small firms [58].

#### H#2b) The most commonly used crisis management measures in HR are linked to the ownership of the organization

Ownership structure also has a significant impact on the HR strategy for pandemic management. In multinational companies, reconciling the interests of foreign owners with those of local management and employees is a recurring problem [59–62]. Foreign owners aim to maximize profits, while in domestically owned firms, operational viability is at least as important [63]. The new environment is characterized by pressures and expectations from different stakeholder groups, heightened expectations of social engagement and corporate membership, and radical uncertainty about the future. In the wake of COVID-19, boards are likely to face increased pressure to incorporate stakeholder perspectives and opinions, particularly those of employees, into their monitoring and decision-making processes.

### H#3: The crisis situation reinforces the practical recognition of the organizational importance of human resources—and some of their layers

The role of HRM in crisis situations such as the current one is clear to researchers [16, 25, 63]. Reducing costs in the short term by ‘sacrificing’ human resources [64, 65] also makes adaptation, long-term growth and recovery from a crisis more difficult. Treating HRM as a strategic function [7, 43] or investing in employee engagement, however, will pay off in crisis situations like the current one [66]. In the third hypothesis, we argue that the importance of HR (the strategic role of HR, the critical resource nature of human capital, the goal-driven nature of retention and development rather than layoffs) has been recognized by practicing HR professionals during the pandemic (increasingly so with time).

### H#4: In addition to increasing the workload for HR, crisis management also increases the demand for professional HR work (H#4a) and offers HR potential for development in a number of areas (H#4b)

The expected increase in the importance of HR activities is also present in the previous hypotheses. The first half of our current, fourth hypothesis differs in that here we assume that this will be accompanied by a practical increase in HR work, and an increased use of HR activities [12, 67, 68]. According to H#4b, responding organizations are able to see the opportunities offered by the crisis to achieve changes in the area of HRM (as well) [69–71]. They can identify real opportunities for improvement and potential in the areas of HR-related activities.

## Materials and methods

### Design and instrument

In the study, we have included the following new subsection on the design of our research.” Our data collection tool was a self-completed electronic questionnaire (https://limesurvey.szie.hu/index.php/649441?newtest=Y&lang=hu). The research did not aim for representativeness; its objective was to obtain quick, exploratory data from as many companies and organisations as possible. Accordingly, the sample was accessible, and the link together with the call were distributed to as many potential respondents (companies and organisations) as possible through the personal and institutional networks, events and publications of the researchers and the supporting organisations. The questionnaires were completed voluntarily by responsible managers of companies. The purpose of the questionnaire and the way in which it would be used were stated in the invitation and in the introductory text of the questionnaire, thus ensuring that participants were informed (= consent). Companies were given the opportunity to indicate whether they would like their company’s name to be included in the research reports, and many of them took advantage of this [72]. At the end of the questionnaire, the following information was requested about the respondent whether he/she was the first HR manager in the organisation, his/her gender, whether he/she had a post-secondary education, and if so, in which field. When completing the questionnaire electronically, it was possible not to answer any or all of these questions.

The questionnaire was tested in three stages before being sent out. First, a first version of the questionnaire was prepared by a narrow team of three, then reviewed by the wider research team, and some invited company managers were invited to complete a pilot version. On this basis, the questionnaire was finalised. Minor changes were made between rounds based on experience, but in the current study we only analyse questionnaires that have remained unchanged.

Availability sampling was used in each of the survey stages, so our data are not appropriate for panel analysis. Direct comparisons between the stages would reveal significant differences in the structure of the samples, rather than the real differences between the stages, due to the different, non-representative samples. Nevertheless, the relatively high sample numbers and the wide range of respondents give us confidence that the detection of differences between samples is still scientifically interesting. It may indicate, if not in a generalizable way, changes that can be verified later by other, more targeted studies.

The ethics of the survey have been approved by the committees of several universities involved in the study (EXACT names of ethics committees), and their certificates have been submitted together with the study”.

### Statistical analysis

All the four hypotheses are tested using the data presented above. To test H#1, we examine whether there is a statistically significant difference in the frequency with which certain HR functions are reported to be important by respondents. Therefore, we first describe the relative frequencies using cross-tabulation, and then we check the differences between these waves with a Chi-square test.

Hypothesis H#2 is tested via ANOVA separately for sub-hypotheses ‘a’ and ‘b’. For H#2a, the averages of the responses for each firm-size category, and for H#2b for the different ownership structure, are compared.

Hypothesis H#3 is examined in a similar way to H#2. We first test the average differences among the pandemic-waves and then among the ownership categories using the ANOVA method. The variables tested here are the answers to the questions about the importance of human resources management.

In the case of our fourth hypothesis, we investigated the change in the expectations for professional HR management, measured by a categorical variable (‘number of tasks assigned to HR’, ‘need for professional HR work’) among data collection periods (H#4a). Using Chi-square test and Cramer’s V, we examined whether there is a significant association between the increase in the amount of HR tasks and the (categorically measured) change in HR efficiency, and the change in the amount of HR tasks and the importance of HR.

To examine H#4b, we analysed the interwave variation of the general question about the extent to which the respondents believe that the pandemic has the potential to trigger positive changes (measured on a scale of 1 to 7). ANOVA/Welch, Kruskal–Wallis and chi-square tests were applied. Respondents were also able to indicate in which HR areas they expected to improve. To test the difference in the latter among the three pandemic-waves, we used a Chi-square tests and a Cramer’s V.

## Results

A total of 1664 responses from organizations (companies and institutions) were processed over the three waves of the virus, during three response periods (Phase 1: 12 June 2020–31 July 2020, Phase 2: 01 August 2020–15 November 2020, Phase 3: 01 March 2021–20 April 2021). Almost one fifth of the responding organizations in the total sample are state or municipality owned, while half are domestic private enterprises. In the first data collection period, the share of public/municipal organizations was even lower (11%) than in the second and third data collection periods. Foreign or mixed ownership firms account for roughly a quarter of respondents in all three periods.

Small enterprises with less than 50 employees make up almost half of the total sample (Table 1), and approximately two-thirds of the sample belongs to the SME sector, while enterprises/organizations with more than 250 employees make up almost a third of the sample. The proportion of the latter is highest in the second survey period, at 36%, compared to 27% and 28% in the first and third phases, respectively.

**Table 1:** Distribution of the sample by organizational size (number of employees).

		Period of data collection			Total
		1^st^ wave	2^nd^ wave	3^rd^ wave	
Employees	The organization does not employ anyone	6.0%	3.1%	2.7%	3.7%
	1–49	48.2%	39.0%	50.6%	43.0%
	50–250	18.7%	21.6%	19.0%	20.5%
	251–500	9.6%	8.8%	7.6%	8.8%
	over 500	17.6%	27.5%	20.2%	24.1%
	Total n = (100%)	386	1010	263	1659
Missing		1	4	0	5
**Total n =**		**387**	**1014**	**263**	**1664**

Source: authors’ own editing

### Testing our hypotheses

In this section each of the four hypotheses will be examined in consecutive subsections.

### H#1: Crisis management measures in the HR area will reposition the importance of individual HR functions

The vast majority of the organizations surveyed do not have measures to reduce labour needs and working hours. Of the measures related to staff management, the only one used more frequently was a staff freeze (49%), while only 37% of the respondents had used working time reduction. The use of both was more prevalent in the first reference period, while in the third phase their use decreased significantly (from 55% to 38%, and from 44% to 29%, respectively). In both cases, there is a significant relationship with the survey period (Chi-square test sig.<0.001). However, the use of teleworking was common, with 76% of organizations making some use of it, and slightly more in the first period (80% vs. 74% and 73%, respectively), as was the development or revision of succession and replacement plans, with almost two thirds of respondents (65%) adopting these strategies. Lay-offs, reducing labour needs and reducing working hours were therefore less common in the firms surveyed (Table 2).

**Table 2:** Use of HR crisis management measures.

HR measures	Typical of			
	1^st^ wave	2^nd^ wave	3^rd^ wave	Total
Nothing to do	38.5%	30.9%	32.9%	32.9%
Staff freeze	55.1%	49.1%	38.0%	48.7%
Staff cuts, downsizing	35.7%	27.3%	23.9%	28.7%
Reduction of leased labour	24.5%	26.3%	20.9%	25.0%
Reduce labour needs through automation/technical solutions	26.9%	29.7%	27.2%	28.7%
Reduce labour needs through training, development	25.1%	28.3%	23.1%	26.8%
Reduction of working time	44.0%	36.7%	29.0%	37.1%
Allowing/ordering home office work	80.4%	74.4%	73.0%	75.5%
Develop/revise succession, replacement plans	61.5%	66.2%	64.8%	64.9%
Wage freezes	38.3%	22.5%	20.1%	25.7%
Wage reduction	22.7%	20.3%	12.5%	19.6%
Reduction of fringe benefits	27.7%	25.8%	16.9%	24.8%
Assisting workers with social problems	68.6%	65.4%	68.6%	66.6%
New health and safety measures	81.6%	85.7%	84.0%	84.5%
Reducing the risk of pandemic through training	44.9%	45.0%	41.4%	44.4%
Support for self-development	57.5%	49.6%	59.1%	52.9%
Revision of the performance appraisal system	30.4%	38.2%	41.4%	37.0%
Revision of the incentive scheme	35.6%	40.6%	41.8%	39.6%
Revision of equal opportunities strategy/plan	18.2%	27.3%	28.9%	25.5%

Source: authors’ own editing

In addition, helping employees with their social problems (67%) and new health and safety measures (85%) were also found to be crisis management measures with a significant level of use in the HR field in both periods under review. However, measures to reduce wages and benefits were only found in about a quarter of organizations in periods 1 and 2, and the proportion of organizations using them fell further in period 3 (to 13% and 17% respectively).

The two most frequently used measures were new health and safety measures (85%) and authorizing/requiring home office work (76%), followed by measures to help workers with social problems (67%) and the development/revision of succession and replacement plans (65%). Of these, there is a significant difference (Chi-squared test sig.<0.001) between the survey periods for the authorization/regulation of home office work, with the highest proportion (80%) for the first measure, while for the other three measures there is no significant difference between the periods.

For the period ahead, *recruitment*, *selection*, *headhunting*, *hiring and workforce management* were identified by the organizations as the HR work areas of increasing importance, and the role of these areas even increased somewhat in the second and third periods. Second was *retention*, *motivation*, *incentive*, *and engagement*, and these showed an increase in the third (2021) data collection period. It can be seen that the organizations surveyed see securing, motivating and retaining the right workforce as an area of increasing importance for HR in the near future. As with crisis management measures, HR work related to reducing labour needs will not be at the forefront in the near future.

In third and fourth place, neck and neck, were *administration*, *labour law*, *labour affairs*, and *internal/personal/online communication*, *liaison*, *and information*, and while the importance of the former group shows a slight increase in the later period compared to the first, the latter shows a decrease. These groups are followed closely in 5^th^ place by *training and development*, *online education*, *and e-learning*. *Home office*, *teleworking*, *and atypical employment* ranked 6^th^. The latter were therefore also among the HR areas whose importance would increase most in the near future, although they were considered less important compared to their role and importance in crisis management measures. *Labour safety*, *health protection*, *occupational health* and *various tasks related to the pandemic* (such as provision of protective equipment, tests, measures, coordination, management of social/psychological problems, etc.) were only ranked 8^th^ and 9^th^, although the related crisis management measures were also predicted to be of greater importance in these areas in the longer term.

*On this basis*, *hypothesis 1 is considered not to be rejected*.

### H#2: The most commonly used crisis management measures in HR are organizational context-dependent

#### H#2a) The crisis management measures used depend on the size of the organization

The average values calculated from the responses on a scale of 1 to 4 (1-not at all; 4-highly characteristic) and the results of the statistical tests carried out are shown in Table 3. There is a significant relationship between HR crisis management measures and organizational size in 15 cases and a clear lack of relationship between the variables in 4 cases. These include measures related to wage freezes, wage and benefit cuts and working time reductions. In all four cases, the sample means are below 2, meaning that organizations are less likely to take advantage of these measures, regardless of the number of their employees. The most striking difference between organizations of different sizes (with different numbers of staff) is the use of the following measures:

- *Authorization/ordering of home office work*
- *New health and safety measures*
- *Development/revision of succession and replacement plans*
- *Reduction of leased labor*
- *Reduction of the risk of the pandemic through training*

**Table 3:** Relationships between HR crisis management actions and organizational size–means and statistical tests.

	Averages						Tests						
	The organis. did not employ anyone	1–49 pple	50–250 pple	251–500 pple	above 500 pple	Overall	Chi Square Sig	Cramer’s V	Homo-scedasti-city	ANOVA Sig	Welch Sig	Kruskal-Wallis Sig	Eta
No tasks	1.81	1.79	1.45	1.45	1.45	1.61	0.000	0.110	0.000	0.000	0.000	0.000	0.172
Zero growth in staff	1.39	1.88	2.02	2.35	2.19	**2.01**	0.000	0.134	0.000	0.000	0.000	0.000	0.162
Downsizing	1.32	1.45	1.50	1.60	1.51	**1.48**	0.001	0.082	0.144	0.206	0.185	0.006	0.061
Downsizing of temporary staff	1.30	1.27	1.54	1.83	1.62	**1.46**	0.000	0.148	0.000	0.000	0.000	0.000	0.209
Reducing labour requirements by automation/ technical solution	1.54	1.31	1.42	1.64	1.62	**1.44**	0.000	0.118	0.000	0.000	0.000	0.000	0.174
Reducing labour requirements by trainings, development	1.47	1.28	1.34	1.56	1.59	**1.40**	0.000	0.130	0.000	0.000	0.000	0.000	0.178
Reduction of working hours	1.64	1.66	1.73	1.77	1.63	**1.68**	0.751	0.042	0.248	0.600	0.624	0.662	0.042
Enabling/directing home offices	2.33	2.33	3.03	3.15	3.14	**2.75**	0.000	0.209	0.000	0.000	0.000	0.000	0.316
Elaboration/replanning of replacement plans	1.70	1.93	2.22	2.48	2.46	**2.16**	0.000	0.154	0.491	0.000	0.000	0.000	0.232
Pay freeze	1.41	1.48	1.55	1.62	1.52	**1.52**	0.301	0.054	0.026	0.423	0.457	0.288	0.049
Pay cuts	1.48	1.40	1.38	1.30	1.29	**1.36**	0.314	0.054	0.000	0.154	0.134	0.194	0.065
Reducing fringe benefits	1.40	1.47	1.54	1.47	1.40	**1.46**	0.083	0.064	0.001	0.375	0.384	0.679	0.052
Addressing employees’ social problems	1.72	2.04	2.19	2.46	2.40	**2.19**	0.000	0.118	0.247	0.000	0.000	0.000	0.176
New occupational health and safety measures	2.06	2.58	3.12	3.28	3.29	**2.91**	0.000	0.205	0.000	0.000	0.000	0.000	0.323
Reducing the risks of the pandemic through training	1.70	1.56	1.76	1.83	2.02	**1.74**	0.000	0.128	0.000	0.000	0.000	0.000	0.188
Supporting personal development	2.06	1.83	1.89	2.10	2.02	**1.92**	0.003	0.079	0.375	0.007	0.008	0.003	0.094
Revising the performance appraisal system	1.54	1.50	1.59	1.83	1.67	**1.59**	0.002	0.081	0.000	0.000	0.001	0.000	0.114
Revising the incentive scheme	1.62	1.56	1.62	1.83	1.68	**1.63**	0.072	0.064	0.068	0.013	0.024	0.009	0.090
Revising the equal opportunities strategic plan	1.46	1.29	1.33	1.51	1.52	**1.38**	0.000	0.101	0.000	0.000	0.000	0.000	0.139

Source: authors’ own editing

All of these measures are more widely used by organizations with larger workforces, and their use is also increasing as the number of staff categories increases, with the exception of temporary leased labor. For organizations with more than 50 employees, the first two measures show high average values of more than 3, which distinguishes them from smaller organizations.

In the case of staff freezes and downsizing, it is only enterprises with no employees that are significantly differentiated from the others, with small enterprises with between 1 and 49 employees having average values just below those of larger enterprises.

Small enterprises with less than 50 employees and enterprises with no employees are more affected by the "do nothing" attitude, although even in their case the average values are still below 2. This suggests that some of these enterprises are also taking some action.

*On this basis, hypothesis 2a) is considered not to be rejected*.

#### H#2b) The most commonly used crisis management measures in HR are linked to the ownership of the organization

The statistical tests carried out (see Table 4) show a significant relationship between HR crisis management measures and the owner in most cases, except for one case: the reduction of fringe benefits, which is hardly ever applied by the organizations surveyed, regardless of ownership (mean value 1.46).

**Table 4:** Relationships between HR crisis management actions and ownership structure–means and statistical tests.

	Averages					Tests						
	Govern-mental, municipial	Domestic	Foreign or Joint Vent.	Non-profit	Overall	Chi Square Sig	Cramer’s V	Homo-scedasti-city	ANOVA Sig	Welch Sig	Kruskal-Wallis Sig	Eta
No tasks	1.57	1.73	1.45	1.47	**1.61**	0.000	0.085	0.000	0.000	0.000	0.001	0.121
Zero growth in staff	1.97	1.89	2.29	1.79	**2.01**	0.000	0.115	0.066	0.000	0.000	0.000	0.148
Downsizing	1.20	1.54	1.60	1.39	**1.48**	0.000	0.101	0.000	0.000	0.000	0.000	0.164
Downsizing of temporary staff	1.25	1.35	1.84	1.32	**1.47**	0.000	0.160	0.000	0.000	0.000	0.000	0.257
Reducing labour requirements by automation/ technical solution	1.35	1.40	1.61	1.36	**1.44**	0.000	0.082	0.000	0.000	0.000	0.000	0.128
Reducing labour requirements by trainings, development	1.32	1.34	1.59	1.25	**1.40**	0.000	0.099	0.000	0.000	0.000	0.000	0.158
Reduction of working hours	1.47	1.73	1.73	1.63	**1.68**	0.009	0.068	0.000	0.001	0.001	0.001	0.100
Enabling/directing home offices	2.77	2.40	3.31	3.07	**2.75**	0.000	0.208	0.000	0.000	0.000	0.000	0.319
Elaboration/replanning of replacement plans	2.41	1.99	2.33	1.98	**2.16**	0.000	0.120	0.011	0.000	0.302	0.000	0.176
Pay freeze	1.36	1.49	1.65	1.66	**1.51**	0.007	0.069	0.000	0.001	0.002	0.000	0.104
Pay cuts	1.20	1.43	1.32	1.58	**1.36**	0.001	0.076	0.000	0.000	0.000	0.000	0.121
Reducing fringe benefits	1.43	1.50	1.41	1.51	**1.46**	0.446	0.043	0.004	0.308	0.302	0.563	0.048
Addressing employees’ social problems	2.16	2.11	2.35	2.11	**2.19**	0.000	0.083	0.000	0.001	0.002	0.002	0.099
New occupational health and safety measures	3.04	2.67	3.28	2.76	**2.91**	0.000	0.145	0.000	0.000	0.000	0.000	0.241
Reducing the risks of the pandemic through training	1.74	1.61	1.99	1.75	**1.74**	0.000	0.114	0.094	0.000	0.000	0.000	0.162
Supporting personal development	1.82	1.87	2.07	1.95	**1.92**	0.024	0.063	0.474	0.002	0.003	0.002	0.095
Revising the performance appraisal system	1.45	1.58	1.71	1.59	**1.59**	0.005	0.070	0.000	0.001	0.001	0.002	0.100
Revising the incentive scheme	1.47	1.64	1.72	1.65	**1.63**	0.014	0.066	0.000	0.003	0.001	0.007	0.094
Revising the equal opportunities strategic plan	1.37	1.33	1.47	1.44	**1.38**	0.035	0.062	0.000	0.010	0.019	0.030	0.084

Source: authors’ own editing

The most marked difference between the various organizational categories is observed for the same measures already seen in relation to the number of employees in the organization:

- *Authorization/ordering of home office work*
- *New health and safety measures*
- *Reduction of leased labor*
- *Development/revision of succession and replacement plans*
- *Reduction of the risk of the pandemic through training*

This is mainly due to the fact that half of the large organizations in the total sample with more than 250 employees belong to the foreign or mixed-ownership sector, another third are public/private institutions, while 84% of the enterprises with no employees and 76% of those with 1–49 employees are in the domestic private sector.

Foreign-owned enterprises are much more likely to use the above measures than domestic ones, and the average scores for the first two of these ownership categories above 3 are also outstanding compared to the other measures. On the other hand, the highest average score (2.41) for the implementation/revision of succession and replacement plans is found in public/private organizations, followed only by foreign-owned enterprises (2.33), with the others lagging significantly behind. It can also be seen that in the case of the ’Nothing to do’ attitude, the average value of 1.73 for the domestic private sector is noticeably higher than for the other types of organizations (Table 4).

It is also worth noting that, in addition to cutting leased labor, staff freezes, and downsizing are more common in foreign-owned enterprises than in others. That is, these measures depend more on ownership than on organizational size (cf. hypothesis 2a).

*On this basis*, *we consider hypothesis 2b) to be maintained*.

### H#3: The crisis situation reinforces the practical recognition of the organizational importance of human resources–and some of their layers

Averaging the responses on a scale of 1 to 4 (1-not at all; 4-highly characteristic) gives the average values shown in Table 5. Average values above 3 indicate that, on average, the organizations surveyed felt that *they consider human resources to be of strategic importance and that retaining key employees and talent is particularly important* to them in the current climate, with around half of respondents in both cases considering them to be highly specific to their own organization. *The unique*, *difficult to replace knowledge and expertise within their organization* is seen as a way out of the crisis to a lesser extent (average: 2.58) (only a quarter of the organizations surveyed considered it to be highly specific to them). The responses are relatively homogeneous for all three questions, with no significant variation in dispersion across the three periods. However, the proportion of responses that are highly specific was higher in all three cases in the first survey and in the subsequent survey samples.

**Table 5:** Importance of organizational knowledge and expertise–means and standard deviations.

	1^st^ wave		2^nd^ wave		3^rd^ wave		Total	
	Means	Std. dev.	Means	Std. dev.	Means	Std. dev.	Means	Std. dev.
Human resources are of strategic importance to our organization (N = 1574)	3.33	1.027	3.11	1.054	3.12	1.093	3.16	1.058
The unique, difficult to replace knowledge and expertise within our organization can provide a way out of the crisis (N = 1558)	2.75	1.094	2.52	1.086	2.61	1.073	2.58	1.089
Redundancies linked to the crisis are damaging in the long term because significant intellectual capital is leaving our organization (N = 1557)	2.05	1.236	2.16	1.216	1.92	1.148	2.10	1.212
Retaining key people and talent has become particularly important for us now (N = 1565)	3.17	1.078	3.07	1.083	3.13	1.047	3.10	1.077
The importance of continuous and well-organized training is of paramount importance in making our organization less affected by the crisis (N = 1561)	2.22	1.124	2.23	1.116	2.13	1.078	2.21	1.112

Source: authors’ own editing

*Continuous training*, on the other hand, is less common among respondents in reducing the impact of the crisis on their organization, with averages of just over 2 per cent, with 36% of respondents saying it is not common. In the perception of the long-term damage to the organization of *crisis-related redundancies and of significant intellectual capital leaving the organization*, the standard deviation values show a more significant difference between responding organizations, with averages of only around 2, while the proportion of responses saying it is not typical is 48%, and there is no significant difference between the proportions for the three data collection periods.

Furthermore, the averages for foreign-owned firms are higher than the others in all cases (Table 6), so they are more representative of each other, and their responses are more homogeneous in most cases. Only in the perception of the *long-term negative impact of crisis-related redundancies* do the responses of foreign-owned firms show a greater dispersion. Except for *the retention of key people and talent*, in the other cases there is a significant relationship with the ownership of the organization (ANOVA/Welch Sig<0.001). In this case, there is no significant difference not only in the means but also in the degree of dispersion of responses between the responses of the different ownership categories, so that this is considered to be quite specific to firms regardless of ownership, with a mean of 3.10 for the whole sample.

**Table 6:** Impact of ownership on the importance of organizational knowledge and expertise–means and standard deviations.

	State or municipal-owned		Domestic private		Foreign or mixed ownership		Non-profit		Total	
	Means	Std. dev.	Means	Std. dev.	Means	Std. dev.	Means	Std. dev.	Means	Std. dev.
Human resources are of strategic importance to our organization (N = 1574)	3.23	0.980	3.00	1.153	3.39	0.854	3.22	1.131	3.16	1.058
The unique, difficult to replace knowledge and expertise within our organization can provide a way out of the crisis (N = 1558)	2.45	1.086	2.55	1.118	2.75	0.997	2.50	1.232	2.58	1.089
Redundancies linked to the crisis are damaging in the long term because significant intellectual capital is leaving our organization (N = 1557)	1.88	1.133	2.12	1.220	2.24	1.230	1.96	1.250	2.10	1.212
Retaining key people and talent has become particularly important for us now (N = 1565)	3.00	1.089	3.09	1.103	3.19	1.006	3.12	1.135	3.10	1.077
The importance of continuous and well-organized training is of paramount importance in making our organization less affected by the crisis (N = 1561)	2.28	1.123	2.05	1.098	2.49	1.069	2.04	1.133	2.21	1.112

Source: authors’ own editing

*On this basis, hypothesis 3 is not rejected (retained)*.

### H#4: In addition to increasing the workload for HR, crisis management also increases the demand for professional HR work and offers HR potential for development in a number of areas

One third of the organizations surveyed do not have a separate HR department or HR function, and among those that do, almost half of the organizations reported an increase in expectations of HR work: 31% of the total sample and 46% of those with HR reported this (Table 7).

**Table 7:** Changes in expectations of the existence of an HR organization and its effectiveness.

N = 1636		Period of data collection			Total
		1^st^ wave	2^nd^ wave	3^rd^ wave	
Expectation of the efficiency of the HR department, HR activities:	There is no separate HR department or function	37.8%	30.5%	35.8%	33.0%
	Decreased	2.9%	1.1%	2.3%	1.7%
	No change	26.0%	39.0%	29.2%	34.5%
	Increased	33.2%	29.4%	32.7%	30.8%
Total		100,0%	100.0%	100.0%	100.0%

Source: authors’ own editing

In organizations with a separate HR department or HR function, more than half of respondents (54%) also reported an increase in the volume of tasks. Increases were prevalent in all three data collection periods and were more pronounced in the first period, affecting almost two-thirds of respondents (Table 8). The Chi-square test showed a significant relationship between the two variables (period of data collection—amount of HR tasks) (Sig. = 0.023).

**Table 8:** Changes in the number of tasks assigned to HR.

N = 1093		Period of data collection			Total
		1^st^ wave	2^nd^ wave	3^rd^ wave	
Volume of HR tasks:	Decreased	6.0%	3.9%	3.0%	4.2%
	No change	32.3%	44.0%	42.8%	41.4%
	Increased	61.6%	52.1%	54.2%	54.4%
Total		100,0%	100.0%	100.0%	100.0%

Source: authors’ own editing

In addition to the increase in the volume of HR tasks, the majority also reported a further increase in the importance of professional HR work, with 51% of respondents overall saying it was moderately or very important, while almost a third of respondents said it was not at all important (Table 9). In this case, the Chi-square test showed no significant relationship between the two variables (Sig. = 0.253).

**Table 9:** Changes in the need for professional HR work.

N = 1562		Period of data collection			Total
		1^st^ wave	2^nd^ wave	3^rd^ wave	
The importance of professional HR work continues to grow	Not typical	30.5%	29.6%	31.0%	30.0%
	Typical to a small extent	15.4%	20.0%	20.6%	19.1%
	Moderately typical	22.8%	24.9%	24.6%	24.4%
	Typical to a great extent	31.4%	25.5%	23.8%	26.5%
Total		100,0%	100.0%	100.0%	100.0%

Source: authors’ own editing

The change in the volume of HR tasks also shows a significant, medium-strong relationship with the expectations (decreasing, unchanged or increasing) of HR department/activity effectiveness (Chi-square test sig<0.001; Cramer’s = 0.557) and with the increasing importance of HR (Chi-square test sig<0.001; Cramer’s = 0.309). For 90% of those who reported an increase in expectations of HR work effectiveness, the amount of HR tasks also increased, while 59% of those who reported a decrease in expectations reported a decrease in tasks, with 22% reporting an increase, and 19% reporting no change in the number of tasks. Among organizations reporting a high level of the increased importance of HR work, 76% reported an increase in the volume of tasks and only 2% reported a decrease in tasks. However, among those where there was no increase in the importance of HR work, 75% reported no change in the volume of work. It can also be observed that as the importance of HR work increases, the proportion reporting an increase in tasks increases and the proportion reporting no change or a decrease in tasks decreases.

As we have already seen in testing Hypothesis 1, *recruitment*, *selection*, *headhunting*, *hiring*, *and staff management* were the HR work areas of increasing importance, followed by *retention*, *motivation*, *incentives*, *and engagement*. *Administration*, *labor law*, *and labour affairs* and *internal/personal/online communication*, *contact*, *and information* were ranked third and fourth, followed closely by *training and development*, *online education*, *and e-learning* in 5^th^ place and *home office*, *teleworking*, *and atypical employment* in 6^th^ place.

In response to the question "*To what extent do you agree that the pandemic/coronavirus crisis is both an opportunity for your organization and a pressure on your organization to make positive changes*?" (Table 10), 11% of the organizations surveyed said that they did not see the pandemic/coronavirus crisis as an opportunity for positive change at all, while 19% said that they saw it as a significant opportunity. Half of the organizations scored 5 or higher, with an average of 4.39 responses, but the high standard deviation values indicate a division of opinion.

**Table 10:** Perception of the COVID-19 pandemic as an opportunity.

N = 1584	Period of data collection			
	1st wave	2nd wave	3rd wave	Total
**1-I do not agree at all**	11.5%	12.4%	7.5%	11.4%
2	7.4%	7.4%	8.3%	7.6%
3	8.9%	13.7%	10.3%	12.1%
4	10.0%	21.0%	17.8%	18.1%
5	20.6%	19.0%	21.7%	19.8%
6	13.8%	10.0%	16.2%	11.8%
**7-I agree entirely**	27.8%	16.4%	18.2%	19.2%
**Means**	4.73	4.22	4.59	4.39
**Std. deviation**	2.037	1.890	1.809	1.923

Source: authors’ own editing

At the time of the first survey, respondents were generally more optimistic, with more than a quarter of them entirely agreeing that the pandemic is a positive opportunity for them, with an average close to 5, while the results of the second survey show a higher level of pessimism (mean: 4.22) and agreement (with a lower standard deviation). The results of the third survey again reflect a growing optimism: compared to 12% previously, only 7% of respondents do not see a positive potential in the pandemic, while the mean of the responses is again higher (4.59). There is a significant relationship between the period of data collection and the perception of potential for improvement (ANOVA/Welch sig<0.001; Kruskal Wallis sig<0.001; Chi-square test sig<0.001).

Most organizations see potential for improvement mainly in the areas of *internal communication* (56%), *atypical employment/home office* (47%) and *occupational health and safety* (44%). The proportion of those indicating atypical employment decreased from 54% to 45% in the second and third surveys, while the proportion indicating health and safety/home office increased from 36% to 47% and 44% respectively, and in both cases, there is a significant relationship with time (Chi-square test sig = 0.010 and 0.001). However, in all three survey samples, the proportion of those indicating internal communication as the most frequent area of choice for most organizations remained virtually unchanged (57% vs. 56%) (Table 11).

**Table 11:** HR-level development opportunities during the three waves.

HR areas	1^st^ wave	2^nd^ wave	3^rd^ wave	Total (n = 1614)
staff planning, succession planning	32.1%	31.7%	31.9%	31.8%
job analysis and planning	31.0%	35.0%	39.3%	34.8%
recruitment, selection and onboarding systems	20.6%	26.7%	26.5%	25.3%
atypical employment/home office work	53.8%	44.6%	45.1%	46.7%
performance management	25.1%	23.0%	26.1%	23.9%
incentive and remuneration management	24.2%	26.9%	25.3%	26.1%
social, mental, family support development	23.9%	24.9%	28.8%	25.3%
human resources development	19.2%	22.4%	24.5%	22.0%
labour relations, participation, inclusion	11.3%	15.7%	12.1%	14.1%
occupational health and safety, health promotion	36.1%	47.3%	43.6%	44.2%
career planning	9.3%	12.4%	12.8%	11.8%
internal communication	57.2%	55.5%	57.2%	56.1%
retention management	28.7%	28.7%	27.6%	28.6%
generation management	11.5%	7.4%	7.0%	8.2%
equal opportunities	5.9%	9.8%	10.1%	9.0%
diversity management	6.5%	6.9%	5.8%	6.6%

Source: authors’ own editing

In addition to these areas, a relatively high number of respondents (around one-third of respondents) also selected the areas of *workforce planning*, *succession planning* and *job analysis and planning*. In the first case, there is no significant difference between the data collection periods, while the second was increasingly selected, with 39% of respondents in the third round identifying it as a potential area for development, compared to 31% in the first period. In addition to the major differences between the three periods already mentioned, there is also a major change in the proportion of respondents who selected *recruitment*, *selection and onboarding systems* (from 21% to 27%). For the other areas, the proportions are surprisingly similar across the three data collection samples.

*On this basis, we also maintain (do not reject) Hypothesis 4*.

## Conclusion

In the world of work, the COVID pandemic is having a real and substantial impact, based on the literature review presented and the analyses carried out. The three waves that have already taken place suggest that this crisis is predominantly associated with a lasting transformation of the workplace. In our view, it differs from the economic crises we have experienced in recent decades. Let us recall: the last global economic crisis, which started in America, in the banking sector, disrupted the daily lives of many people, not only in the financial institutions themselves, but also through mortgage financing; however, after a few years we were back to normal.

Among the scenarios outlined by [41–51], in our paper, our hypotheses and results support the third scenario, the ’emergence of a new normal’ (Let’s strive for a new and better normal). Our empirical data from the first three waves point towards the emergence of such a ’new normal’ in the world of work and can also be used as a toolkit for recovery from the crisis. The spread of home office–hybrid working, the rise of online business negotiations, and paperless–electronic administration, are all associated with a complex digitalization of the world of work, which fits into the emerging concepts of the platform economy [73, 74], on the one hand, and also with the discourse that workplace diversity provides a competitive advantage over familiar workplace monocultures [75].

This is accompanied by new procedures, new norms and new skills on the part of employers and employees alike. Our research and analysis support this.

Among the crisis management measures, the firms we studied preferred working from home, introducing new work and health protection measures, and addressing workers’ social and health problems, rather than the classical redundancies, working time reductions, wage cuts or phasing out fringe benefits. Our Hypothesis 1 supports this.

Moreover, our results presented in Hypotheses 2a and 2b suggest that the adoption of home office and the introduction of new health and safety measures are more common in organizations with more than 50 employees and in foreign-owned organizations. This, in turn, may push the whole Hungarian labor market towards a "new normal" in the medium term by introducing new procedures and new standards in larger and more complex workplaces.

The results presented in Hypotheses 3 and 4 suggest that the HR field could emerge from the pandemic stronger, which could induce a qualitative change in industrial relations. Our research data suggest that while downsizing was unavoidable in many sectors, the retention of key people and talent was accompanied by above average efforts. In addition, the data presented in Hypothesis 3 suggest that the importance of the HR strategy is itself reinforced by the impact of the COVID crisis. Our data also suggest that there has been a significant change in the volume of HR work: the volume of tasks in the HR area has increased significantly, and expectations have also increased. As a consequence, as presented in Hypothesis 4, professionalism becomes particularly important, reinforcing the moves towards a ’new normal’ as discussed so far.

**Fig 1:**
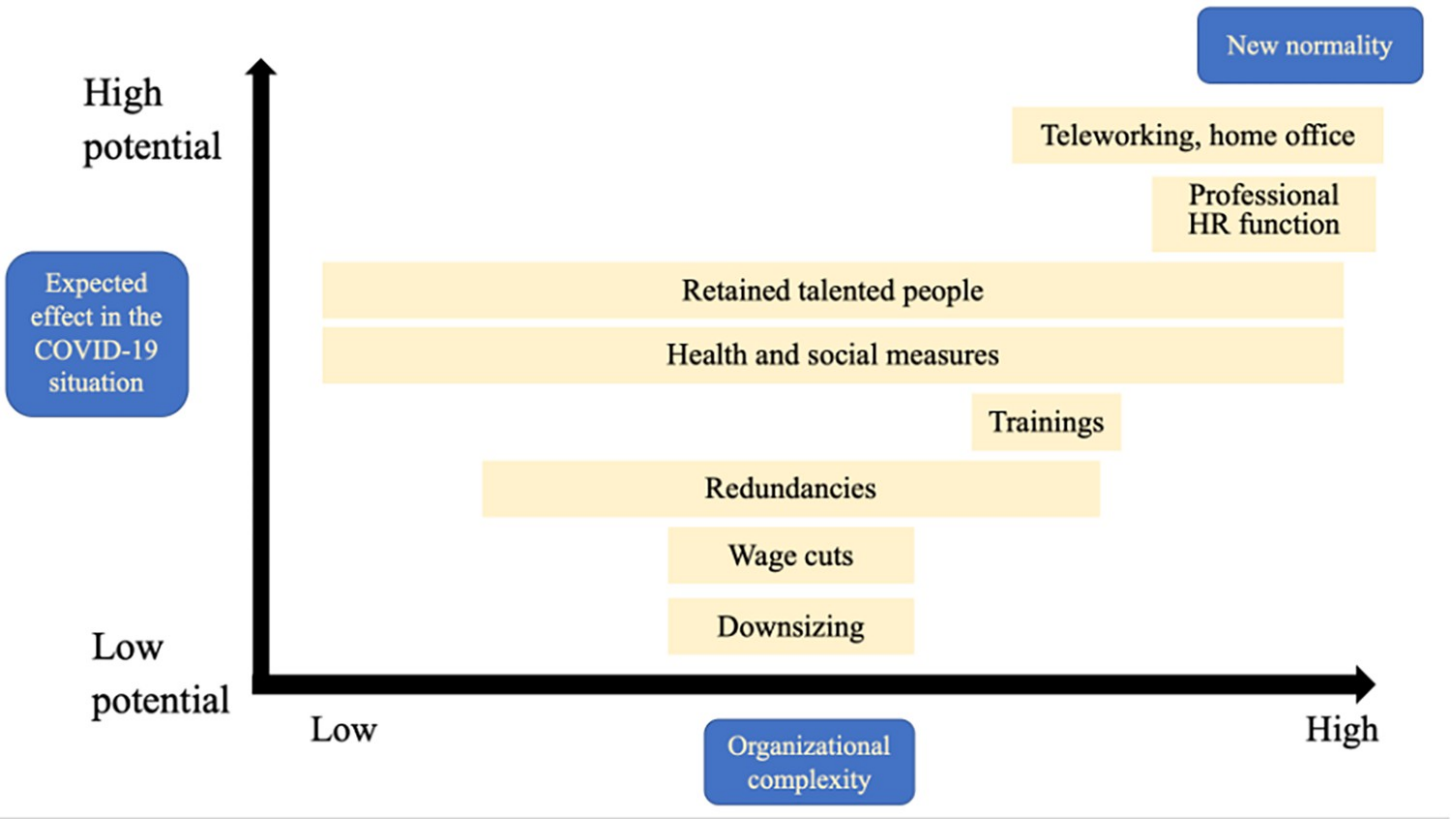
Possible dimensions of the emergence of a "new normal" in the world of work. Source: authors’ own editing.

The research findings presented here project the emergence of a ’new normal’ in the potential dimensions illustrated in Fig 1.

In thinking about the "new normal" in value creation in organizations, we believe that after the pandemic, there is no way around rethinking a "people-centered approach" alongside the traditional efficiency and cost-benefit approach. Indeed, in the context of the pandemic, in addition to the availability of labor as a resource, several dimensions of the issue of availability have come to the fore in terms of competitiveness (see, for example, the issue of family quarantine or even the issue of working time frames for the home office).

### Limitations and future directions of the research

Our research was based on our global literature review and was conducted in 2020 and the first half of 2021 in Hungary, a country in Central Europe. Consequently, one of the possible limitations of our research is the question of whether the HRM experience of a small country can be an example for people living and working in other countries. The recent human crisis has shaken our whole world and highlighted the interconnectedness of nations and peoples [76, 77]. Another limitation is that our three empirical studies conducted over three waves are not representative. However, most of the respondents in our three datasets were dominant organizations and their industry distribution and size reflect the situation in the Hungarian economy. In parallel to the research described in this paper, a similar survey was carried out in five other Central and Eastern European countries (Austria, Bosnia and Herzegovina, Bulgaria, Romania and Slovakia) using the questionnaire presented in this paper and is currently being evaluated. In another empirical study, we looked at the way in which the economies of Hungary and neighboring Slovakia bounced back from the COVID-19 crisis in 2021. The evaluation of the experience of this latter research is also ongoing.

However, the process of recovery from the COVID-19 crisis and the initial recovery could be overshadowed by a new crisis, which could be triggered by the Russian-Ukrainian conflict [78]. The war in Europe has already triggered global economic effects within two months, currently culminating in rapidly rising inflation in Europe, as well as in the shortage of raw materials and increased uncertainty.

## References

[1] Ulrich D, Allen J, Brockbank W, Younger J, Nyman M. HR Transformation. Maidenhead: McGraw-Hill; 2009.

[2] Armstrong M, Taylor S. Armstrong’s handbook of human resource management practice. USA: Kogan Page Publishers; 2020.

[3] Carnevale JB, Hatak I. Employee adjustment and well-being in the era of COVID-19: Implications for human resource management. Journal of Business Research. 2020;116: 183–187. doi: 10.1016/j.jbusres.2020.05.037 32501303 PMC7241356

[4] Pradhan RK, Jandu K, Panda M, Hati L, Mallick M. In pursuit of happiness at work: exploring the role of psychological capital and coping in managing COVID-19 stress among Indian employees. Journal of Asia Business Studies 2021; Vol. ahead-of-print No. ahead-of-print. ISSN: 1558-7894. doi: 1108/JABS-03-2021-0097

[5] Gonçalves PS, dos Santos JV, Silva AI, Veloso A, Brandão C, Moura R. COVID-19 and People Management: The View of Human Resource Managers. Administrative Sciences. 2021;11(69): 1–13.

[6] Harbert T. The Pandemic Has Expanded the Role of HR. 2021;09: 01. Available from: https://www.shrm.org/hr-today/news/hr-magazine/fall2021/pages/pandemic-expands-role-of-hr.aspx

[7] Bingham S. How HR Leaders Can Adapt to Uncertain Times. Harvard Business Review. 2020 Aug 4 [Cited 2020 Dec 10]. Available from: https://hbr.org/2020/08/how-hr-leaders-can-adapt-to-uncertain-times.

[8] Komm A, Pollner F, Schaninger B, Sikka S. The new possible: How HR can help build the organization of the future. McKinsey Global Publishing. 2021;3: 12. Available from: https://www.mckinsey.com/business-functions/people-and-organizational-performance/our-insights/the-new-possible-how-hr-can-help-build-the-organization-of-the-future.

[9] WHO 2020. Attacks on health care in the context of COVID-19. World Health Organization. 2020;7: 30. Available from: ss://www.who.int/news-room/feature-stories/detail/attacks-on-health-care-in-the-context-of-covid-19.

[10] Knobler LS, Mack A, Mahmoud A, Lemon MS. The threat of pandemic influenza. Are we ready? Washington: The National Academic Press; 2005 20669448

[11] CRS. Global Economic Effects of COVID-19. CRS (Congressional Research Service). 2021;11: 10. Available from: https://sgp.fas.org/crs/row/R46270.pdf.

[12] Caligiuri P, De Cieri H, Minbaeva D, Verbeke A, Zimmermann A. International HRM insights for navigating the COVID-19 pandemic: Implications for future research and practice. Journal of International Business Studies. 2020;51: 697–713. doi: 10.1057/s41267-020-00335-9 32836500 PMC7266413

[13] ILO. The impact of the COVID-19 pandemic on jobs and incomes in G20 economies. International Labour Organization (ILO) and OECD, Geneva; 2020. Available from: https://www.ilo.org/wcmsp5/groups/public/—dgreports/—cabinet/documents/publication/wcms_756331.pdf

[14] Shaikh A. The First Great Depression of the 21st Century. Socialist Register. 2011;47: 44–63.

[15] Landivar LC, Ruppanner L, Scarborough WJ, Collins C. Early Signs Indicate That COVID-19 Is Exacerbating Gender Inequality in the Labor Force. Socius: Sociological Research for a Dynamic World. 2020;6: 1–3. doi: 10.1177/2378023120947997 34192138 PMC7399570

[16] Butterick M, Charlwood A. HRM and the COVID‐19 pandemic: How can we stop making a bad situation worse? Human Resource Management Journal. 2021;31: 847–856. doi: 10.1111/1748-8583.12344

[17] Zacher H, Rudolph CW. Researching employee experiences and behavior in times of crisis: Theoretical and methodological considerations and implications for human resource management. German Journal of Human Resource Management: Zeitschrift für Personalforschung. 2022;36(1): 6–31. doi: 10.1177/23970022211058812

[18] Adams-Prassl A, Boneva T, Golin M, Rauh C. Inequality in the impact of the coronavirus shock: Evidence from real time surveys. Journal of Public Economics. 2020;189: 104245, doi: 10.1016/j.jpubeco.2020.104245

[19] OECD. OECD Economic Outlook, Volume 2020 Issue 1. OECD. 2020;6: 10. Available from: https://www.oecd-ilibrary.org/economics/oecd-economic-outlook/volume-2020/issue-1_0d1d1e2e-en. doi: 10.1787/0d1d1e2e-en

[20] Bank World. The Global Economy: on Track for Strong but Uneven Growth as COVID-19 Still Weighs. Washington: International Bank for Reconstruction and Development. The World Bank. 2021;6: 8. Available from: https://www.worldbank.org/en/news/feature/2021/06/08/the-global-economy-on-track-for-strong-but-uneven-growth-as-covid-19-still-weighs.

[21] Fazekas K, Csillag M, Hermann Z, Scharle Á. The Hungarian Labour Market 2019. Budapest: Institute of Economics; 2021.

[22] MTI. Majdnem 10 ezer vállalat vette igénybe a munkahelyvédelmi bértámogatást. HR Portál. 2020;5: 25.Available from: https://www.hrportal.hu/c/majdnem-10-ezer-vallalat-vette-igenybe-a-munkahelyvedelmi-bertamogatast-20200526.html.

[23] MTI. Naponta több ezer kérelem érkezik a munkahelyvédelmi bértámogatásra. HR Portál. 2020;6: 19. Available from: https://www.hrportal.hu/hr/naponta-tobb-ezer-kerelem-erkezik-a-munkahelyvedelmi-bertamogatasra-20200619.html.

[24] Spencer DA. Economics and ‘bad’ management: The limits to performativity. Cambridge Journal of Economics. 2020;44(1): 17–32. doi: 10.1093/cje/bez033

[25] Stuart M, Spencer DA, McLachlan CJ, Forde C. COVID‐19 and the uncertain future of HRM: Furlough, job retention and reform. Human Resource Management Journal. 2021;31: 904–917. doi: 10.1111/1748-8583.12395

[26] Gursoy D, Chi CG. Effects of COVID-19 pandemic on hospitality industry: Review of the current situations and a research agenda. Journal of Hospitality Marketing & Management. 2020;29(5): 527–529. doi: 10.1080/19368623.2020.1788231

[27] Kashif M, Rehman AU, Javed K. Restaurants and Covid-19. International Journal of Medical Science in Clinical Research and Review. 2020;3(3): 281–289.

[28] Tirachini A, Cats O. COVID-19 and Public Transportation: Current Assessment, Prospects, and Research Needs. Journal of Public Transportation. 2020;22(1): 1–21. doi: 10.5038/2375-0901.22.1.1 36118518 PMC9468467

[29] Harris J, Sunley P, Evenhuis E, Martin R, Pike A, Harris R. The Covid-19 crisis and manufacturing: How should national and local industrial strategies respond? Local Economy: The Journal of the Local Economy Policy Unit. 2020;35(4): 403–415. doi: 10.1177/0269094220953528

[30] Albitar K, Gerged AM, Kikhia H, Hussainey K. Auditing in times of social distancing: The effect of COVID-19 on auditing quality. International Journal of Accounting & Information Management. 2020;29(1): 169–178. doi: 10.1108/IJAIM-08-2020-0128

[31] MSZÉSZ. 2020. A hazai és nemzetközi szállodaipar teljesítményéről—2020 április. Magyar Szállodák és Éttermek Szövetsége. 2020;6. 10. Available from: http://www.hah.hu/files/1215/9169/2329/Trendriport_2020._prilis.pdf.

[32] Kwan SH. Market Assessment of COVID-19. FRBSF Economic Letter, 2020;14: 1–5.

[33] Lesi H. The Influence of Information Technology Covid-19 Plague Against Financial Statements and Business Practices. Ilomata International Journal of Tax & Accounting. 2020;1(3): 10.

[34] Pantelimon FV, Georgescu TM, Posedaru BS. The Impact of Mobile E-Commerce on GDP: A Comparative Analysis between Romania and Germany and how Covid-19 Influences the e-Commerce Activity Worldwide. Informatica Economica. 2020;24(2/2020): 27–41. doi: 10.24818/issn14531305/24.2.2020.03

[35] Kim PS. South Korea’s fast response to coronavirus disease: Implications on public policy and public management theory. Public Management Review. 2020;23(12): 1–12. doi: 10.1080/14719037.2020.1766266

[36] Taleb NN. The Black Swan: The Impact of the Highly Improbable. The New York Times 2007 April 22 [Cited 2007 May 3]. Available from: https://www.nytimes.com/2007/04/22/books/chapters/0422-1st-tale.html.

[37] Taleb NN. Ten Principles for a Black Swan Robust World. Edge. 2009;4: 7. Available from: https://www.edge.org/conversation/nassim_nicholas_taleb-ten-principles-for-a-black-swan-robust-world.

[38] Agius RM., Robertson JFR, Kendrick D, Sewell HF, Stewart M, McKee M. Covid-19 in the workplace. British Medical Journal. 2020;220(370): m3577. doi: 10.1136/bmj.m3577 32958470

[39] Rodríguez-Barranco M, Rivas-García L, Quiles JL, Redondo-Sánchez D, Aranda-Ramírez P, Llopis-González J, et al. The spread of SARS-CoV-2 in Spain: Hygiene habits, sociodemographic profile, mobility patterns and comorbidities. Environmental Research. 2021;192: 110223. doi: 10.1016/j.envres.2020.110223 32971081 PMC7505892

[40] World Bank. Global Economic Prospects-World Bank Flagship Report. Washington: International Bank for Reconstruction and Development. The World Bank. 2021;6: 2. Available from: https://thedocs.worldbank.org/en/doc/600223300a3685fe68016a484ee867fb-0350012021/original/Global-Economic-Prospects-June-2021.pdf.

[41] Brammer S, Branicki L, Linnenluecke KM. COVID-19, Societalization and the Future of Business in Society. Academy of Management Perspectives. 2019;34(4): 1–38. doi: 10.5465/amp.2019.005

[42] Han E, Tan MMJ, Turk E, Sridhar D, Leung GM, Shibuya K, et al. Lessons learnt from easing COVID-19 restrictions: an analysis of countries and regions in Asia Pacific and Europe. Lancet. 2020;396(10261): 1525–1534. doi: 10.1016/S0140-6736(20)32007-9 32979936 PMC7515628

[43] Arosha S, Adikaram SA, Priyankara HPR, Naotunna NPGSI. Navigating the Crises of COVID-19: Human Resource Professionals Battle Against the Pandemic. South Asian Journal of Human Resources Management. 2021;8(2): 192–218.

[44] Bourgeault IL, Maier CB, Dieleman M, Ball J, MacKenzie A, Nancarrow S, et al. The COVID-19 pandemic presents an opportunity to develop more sustainable health workforces. Human Resources for Health. 2020;18(83): 1–8. doi: 10.1186/s12960-020-00529-0 33129313 PMC7602762

[45] Obrenovic B, Du J, Godinic D, Baslom MMM, Tsoy D. The Threat of COVID-19 and Job Insecurity Impact on Depression and Anxiety: An Empirical Study in the USA. Frontier Psychology. 2021;12: 1–15. doi: 10.3389/fpsyg.2021.648572 34484024 PMC8411708

[46] ILO. Teleworking during the COVID-19 pandemic and beyond A Practical Guide. International Labour Organization (ILO), Geneva; 2020. Available from: https://www.ilo.org/wcmsp5/groups/public/—ed_protect/—protrav/—travail/documents/instructionalmaterial/wcms_751232.pdf.

[47] Chen Z. Influence of Working from Home During the COVID-19 Crisis and HR Practitioner Response. Frontier Psychology. 2021;12: 1–8. doi: 10.3389/fpsyg.2021.710517 34630219 PMC8495417

[48] OECD. An assessment of the impact of COVID-19 on job and skills demand using online job vacancy data. OECD. 2021;4: 9. Available from: https://read.oecd-ilibrary.org/view/?ref=1071_1071334-wh692jshet&title=An-assessment-of-the-impact-of-COVID-19-on-job-and-skills-demand-using-online-job-vacancy-data.

[49] Gigauri I. Influence of Covid-19 Crisis on Human Resource Management and Companies’ Response: The Expert Study. International Journal of Management Science and Business Administration, 2020;6(6): 15–24.

[50] Byrd C.M. Development of Critical Consciousness Competencies in Diversity Courses. 2021; Paper presented at the APA Division 45 Research Conference.

[51] Williams C. COVID-19 and Undeclared Work: Impacts, Challenges and Policy Responses. SSRN Electronic Journal. 2020:1–49. doi: 10.2139/ssrn.3672437

[52] Lositska MT, Bieliaieva N. COVID-19 vaccine refusal, UK Health (2020). HR Crisis Management at Trade Enterprises. EUREKA: Social and Well-Being. 2021;1(1):10–15.

[53] Alves JC, Lok TC, Luo Y, Hao W. Crisis Management for Small Business during the COVID-19 Outbreak: Survival. Resilience and Renewal Strategies of Firms in Macau. Frontiers of Business Research in China. 2020;14(26): 1–29. doi: 10.21203/rs.3.rs-34541/v1

[54] Kirby S. 5 ways COVID-19 has changed workforce management. World Economic Forum. 2020;6: 2. Available from: https://www.weforum.org/agenda/2020/06/covid-homeworking-symptom-of-changing-face-of-workforce-management/.

[55] Petzer M. Coronavirus and the workforce: How can we limit redundancies? Chartered Institute of Personnel and Development (CIPD). 2020; 4: 2. Available from: http://researchonline.ljmu.ac.uk/id/eprint/12960/1/coronavirus-workforce-redundancies_20200402T170659.pdf.

[56] Watkins M. D., &Yaziji M. COVID-19: People and organizations under pressure. 2020; Available from: https://www.imd.org/research-knowledge/articles/COVID-19-under-pressure/

[57] Gregurec I, Furjan MT, Tomičić-Pupek K. The Impact of COVID-19 on Sustainable Business Models in SMEs. *Sustainability*. 2021;13(3): 1098–1121. doi: 10.3390/su13031098

[58] Medici A. How company size is shaping employer Covid-19 protocols. Tampa Bay Business Journal. 2021;8: 25. Available from: https://www.bizjournals.com/tampabay/news/2021/08/25/covid-19-vaccine-mask-mandate-work-employer.html.

[59] Caprar VD. Foreign locals: A cautionary tale on the culture of MNC local employees. Journal of International Business Studies. 2011;42(5): 608–662.

[60] Dowling PJ, Festing M, Engle AD. International Human Resource Management. 7th ed. Andover, UK: Cengage; 2017.

[61] Dundon T, Rafferty A. The (potential) demise of HRM? Human Resource Management Journal. 2018;28(3): 377–391. doi: 10.1111/1748-8583.12195

[62] Festing M, Kraus AS. The impact of the COVID-19 pandemic on global employees. Berlin: ESCP Research Institute of Management (ERIM); 2020.

[63] Collings DG, McMackin J, Nyberg AJ, Wright PM. Strategic Human Resource Management and COVID‐19: Emerging Challenges and Research Opportunities. Journal of Management Studies. 2021;58(5):1378–82. doi: 10.1111/joms.12695

[64] Trevor CO, Nyberg AJ. Keeping Your Headcount When All About You Are Losing Theirs: Downsizing, Voluntary Turnover Rates, and The Moderating Role of HR Practices. Academy of Management Journal. 2008;51(2): 259–276. doi: 10.5465/amj.2008.31767250

[65] Guthrie JP, Datta DK. Dumb and Dumber: The Impact of Downsizing on Firm Performance as Moderated by Industry Conditions. Organization Science. 2008;19(1): 108–123. doi: 10.1287/orsc.1070.0298

[66] Chang E., Chin H, Lee JW. Pre-crisis commitment human resource management and employees’ attitudes in a global pandemic: The role of trust in the government. Human Resource Management. 2021;61 373–387. doi: 10.1002/hrm.22097

[67] Hutchins HM, Wang J. Organizational Crisis Management and Human Resource Development: A Review of the Literature and Implications to HRD Research and Practice. Advances in Developing Human Resources. 2008;10(3): 310–330. doi: 10.1177/1523422308316183

[68] Lee GOM, Warner M. Epidemics, labour markets and unemployment: The impact of SARS on human resource management in the Hong Kong service sector. International Journal of Human Resource Management. 2005;16(5): 752–771. doi: 10.1080/09585190500083202

[69] Ulmer RR, Sellnow TL. Crisis management and the discourse of renewal: Understanding the potential for positive outcomes of crisis. Public Relations Review. 2002;28(4): 361–365. doi: 10.1016/S0363-8111(02)00165-0

[70] Liu Y, Lee JM, Lee C. The challenges and opportunities of a global health crisis: the management and business implications of COVID-19 from an Asian perspective. Asian Business and Management. 2020;19(3): 277–297. doi: 10.1057/S41291-020-00119-X PMC7216126 40476954

[71] Adikaram AS, Priyankara HPR, Naotunna NPGSI. Navigating the Crises of COVID-19: Human Resource Professionals Battle Against the Pandemic. South Asian Journal of Human Resources Management. 2021;8(2): 192–218. doi: 10.1177/23220937211018021

[72] Poór J, Dajnoki K, Jarjabka Á, Pató G. Sz B., Szabó Sz, Szabó K., et al. (szerk.). Koronavírus-válság kihívások és HR válaszok. Első–második–harmadik hullám összehasonlítása. Gödöllő: Magyar Agrár- és Élettudományi Egyetem; 2021.

[73] Cs Makó, Illéssy M, Pap J. Munkavégzés a platformalapú gazdaságban. A foglalkoztatás egy lehetséges modellje? Közgazdasági Szemle. 2020;67(11): 1112–1129.

[74] Greber C, Krzywdzinski M. Brave new digital work? New forms of performance control in crowdwork. In: Vallas S, Kovalainen A. Work and labour in the digital age. Bingley: Emerald Publishing; 2019. pp. 121–144.

[75] Zádori I, Nemeskéri Zs, Szabó Sz. Deglobalizáció vagy reglobalizáció? Munkaerőpiac a vírus előtt, alatt és után. Új Munkaügyi Szemle. 2020;1(3): 2–13.

[76] Budhwar P, Cumming D. New Directions in Management Research and Communication: Lessons from the COVID‐19 Pandemic. British Journal of Management. 2020;31(3): 441–443.

[77] Torbay R. We Must Embrace Our Interconnectedness. Health Affair. 2021;40(3): 544. doi: 10.1377/hlthaff.2021.00014 33646869

[78] Tooze A. Ukraine’s War Has Already Changed the World’s Economy. Foreign policy. 2022;4: 5. Available from: https://foreignpolicy.com/2022/04/05/ukraine-russia-war-world-economy/.

